# Molecular and Morpho-Agronomical Characterization of Root Architecture at Seedling and Reproductive Stages for Drought Tolerance in Wheat

**DOI:** 10.1371/journal.pone.0156528

**Published:** 2016-06-09

**Authors:** Ram Sewak Singh Tomar, Sushma Tiwari, Vinod, Bhojaraja K. Naik, Suresh Chand, Rupesh Deshmukh, Niharika Mallick, Sanjay Singh, Nagendra Kumar Singh, S. M. S. Tomar

**Affiliations:** 1 National Research Centre on Plant Biotechnology, Indian Agricultural Research Institute, Pusa Campus, New Delhi, 110012, India; 2 Division of Genetics, Indian Agricultural Research Institute, Pusa Campus, New Delhi, 110012, India; 3 School of life Sciences, Devi Ahilya Vishwa Vidyalaya, Khandwa Road, Indore, 452017, India; 4 Departement de Phytologie, University Laval, Quebec, QC, G1V0A6, Canada; 5 Directorate of Wheat Research, Karnal, Haryana, 132001, India; Aberystwyth University, UNITED KINGDOM

## Abstract

Water availability is a major limiting factor for wheat (*Triticum aestivum* L.) production in rain-fed agricultural systems worldwide. Root architecture is important for water and nutrition acquisition for all crops, including wheat. A set of 158 diverse wheat genotypes of Australian (72) and Indian (86) origin were studied for morpho-agronomical traits in field under irrigated and drought stress conditions during 2010–11 and 2011-12.Out of these 31 Indian wheat genotypes comprising 28 hexaploid (*Triticum aestivum* L.) and 3 tetraploid (*T*. *durum*) were characterized for root traits at reproductive stage in polyvinyl chloride (PVC) pipes. Roots of drought tolerant genotypes grew upto137cm (C306) as compared to sensitive one of 63cm with a mean value of 94.8cm. Root architecture traits of four drought tolerant (C306, HW2004, HD2888 and NI5439) and drought sensitive (HD2877, HD2012, HD2851 and MACS2496) genotypes were also observed at 6 and 9 days old seedling stage. The genotypes did not show any significant variation for root traits except for longer coleoptiles and shoot and higher absorptive surface area in drought tolerant genotypes. The visible evaluation of root images using WinRhizo Tron root scanner of drought tolerant genotype HW2004 indicated compact root system with longer depth while drought sensitive genotype HD2877 exhibited higher horizontal root spread and less depth at reproductive stage. Thirty SSR markers were used to study genetic variation which ranged from 0.12 to 0.77 with an average value of 0.57. The genotypes were categorized into three subgroups as highly tolerant, sensitive, moderately sensitive and tolerant as intermediate group based on UPGMA cluster, STRUCTURE and principal coordinate analyses. The genotypic clustering was positively correlated to grouping based on root and morpho-agronomical traits. The genetic variability identified in current study demonstrated these traits can be used to improve drought tolerance and association mapping.

## Introduction

Wheat (*Triticum aestivum* L.) is one of the most important crops of the world. India has largest wheat cultivation area (29.50Mha) while its production (95.6mt in 2014) is second after China in world [1]. The higher production was due to favorable environment ensured by water, fertilizer and proper management in India. However, yields under rainfed (dry weather conditions with light intermittent rains or no rains during the crop season) conditions are still low with a productivity level of 1.7th^-1^ [1]. Water scarcity timing and severity play crucial role in deciding the amount of yield loss under rain-fed conditions. To minimize the water scarcity effects, genotypes with improved tolerance to water limitation having better potential need to be grown to achieve maximum yield [2,3]. Progress in developing high yielding drought tolerant cultivars suitable for rain-fed conditions has been slow due to difficulties encountered in direct selection with a significant impact of genotype x environment (GXE) interactions. Lack of proper understanding of drought tolerance mechanisms and complexities in mode of inheritance, breeding of suitable drought tolerant cultivars is not easy. Because, the selection for yield *per se* through empirical plant breeding, is in direct relation to genotypic adaptation to variation in seasonal rainfall, its quantity and distribution [4,5]. Also the understanding of physiological parameters and their integration with traditional selection method is indispensable to better improvement in yield rate per unit area [6,7].

Conventional breeding methodology based on genetic manipulation of plant architecture by both shoot and root systems contribute towards yield enhancement [8]. In pursuit of breeding for drought tolerance, several shoot related physiological traits have been identified and used as selection criteria [9,10]. Root systems essential for absorption of water and nutrients from soil plays a major role in increasing sink size [11]. However, the root system growth and function, efficient root screening methods (root biomass and root length) and phenotyping of root traits have not been studied at length [12,13]. Several methods including phenotyping in hydroponics [14], in soil using clear boxes [15], through wax barriers [16] and in pots or columns of soil [17] have been suggested in different crops. Similar studies targeting root traits in wheat have been conducted but the results were not correlated with yield related traits under field conditions [18]. The changes during plant life cycle in growth and development are responsible for distinctness in the root traits at seedling stage and reproductive stage. In one complete life cycle it undergoes changes from seedling stage to vegetative stage, tiller stage to grain filling stage i.e. reproductive stage. Atta *et al*. [19], reported genetic variability for root length, root diameter and root length density in a set of 15 wheat genotypes grown in field.

Molecular markers have been applied to characterize cultivars independent of plant tissue or environmental effect and cultivar identification at very early stage of plant development [20]. Genetic diversity in the wheat has been examined using PCR-based molecular markers, such as Amplified Fragment Length Polymorphisms, AFLPs [21] and Sequence Tagged Microsatellite Sites (STMSs or, more generally, SSRs) [22] and cpSSRs [23]. The efficiency of polymorphism detection by SSR in wheat is high due to co-dominant nature, chromosome-specific, reproducibility and high information content [24]. The information on genotypic diversity at molecular level and root architecture, shoot characteristics in relation to yield parameters among drought tolerant wheat genotypes is scanty. The present investigation was carried out to study root architecture among drought tolerant and sensitive wheat genotypes at seedling stage under ambient conditions and reproductive stage under drought stress conditions in PVC pipes. Correlation analysis was also done using morphological and agronomical characters in field under drought stress conditions. SSR profiling to determine phylogenetic relationship was also examined among 31 genotypes of wheat (*Triticum aestivum* L. and *T*. *durum* Desf.).

## Materials and Methods

### Plant Material and Growth Conditions

**Fig 1:**
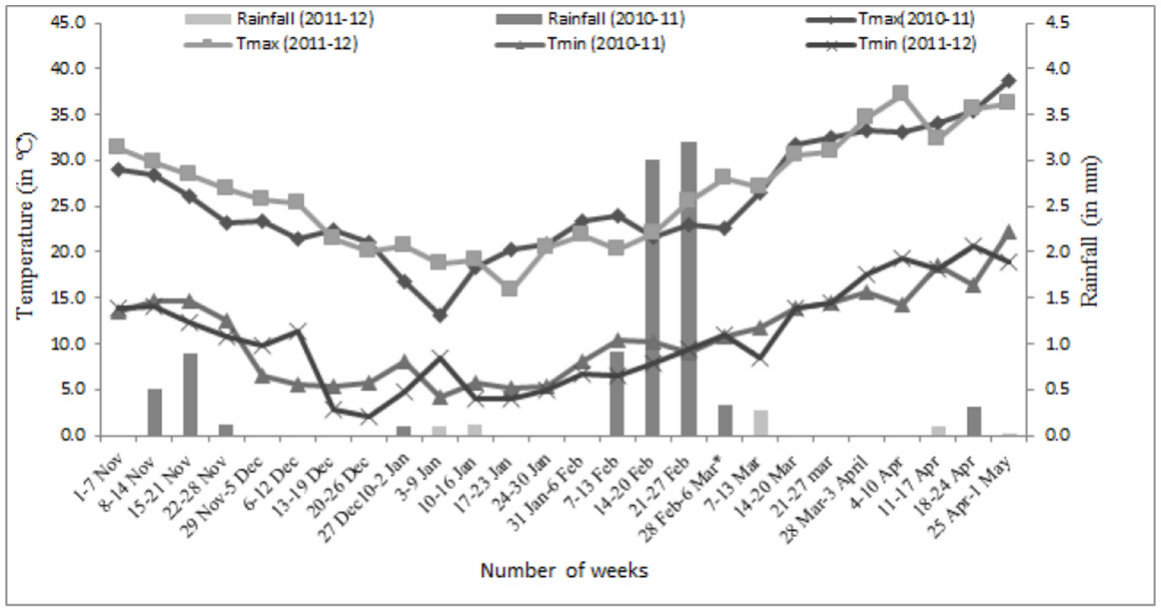
Weekly average of weather conditions during the wheat growth period in 2010–11 and 2011–12. Rainfall for the given period is represented by vertical bars.* indicates average of 8 days in the period from 28^th^ February to 6^th^ March in the year 2011–12.

The present study was done on 158 genotypes of Australian (72) and Indian (86) origin for the diversity analysis recorded on morpho-agronomical traits in field under irrigated and drought stress conditions during 2010–11 and 2011–12 at Indian Agricultural Research Institute, New Delhi (S1 Table). The experiment was conducted in the field of IARI farm located at the altitude of 228.61m above mean sea level (28°38’23” N—77°09’27” E). The soil textures in the field are sandy, loamy and non-calcareous. The value of organic carbon %, pH and electrical conductivity at 0–15cm soil depth were 4.9g/kg,7.9and0.35dsm^-1^, respectively. The average amount of the rainfall, maximum and minimum temperature (Fig 1), relative humidity, wind speed and evaporation rate of each week during the crop growing season for 2010–11 and 2011–12 is presented in S2 Table.

The soil moisture content was determined by using gravimetric method as: Moisture content (%) = Weight of wet soil-weight of dry soil/weight of dry soil x 100. The soil moisture content under rainfed conditions was 24.3% (0–15 cm soil depth) at sowing time during 2010–11. The soil moisture content under vegetative, reproductive and maturity stages was 16.1% and21.4%; 14.2% and 18.2%; 11.9% and 13.6% at 0–15 and 15–30 cm soil depth, respectively. However, during 2011-12it was 23.9% at sowing time while 16.3% and 20.9%, 14.6% and 17.8%and 11.7% and 13.2% at 0–15 and 15–30cm soil depth at vegetative, reproductive and maturity stages, respectively. Out of 158, thirty one Indian wheat genotypes included 28 hexaploid (*T*. *aestivum*) and 3 tetraploid (*T*. *durum*) were used in this study for molecular and root traits characterization (S3 Table). These genotypes have been developed at various Universities or research institutes of the National Agricultural Research System and released for commercial cultivation in different agro-climatic zones of India since 1900 (S3 Table). In the present study, genotypes were selected from the released cultivars for rainfed and irrigated conditions from different wheat growing areas of the country. These genotypes are released only after rigorous testing over the years and locations under moisture stress and irrigated conditions in All India Wheat Coordinated trials prior to their commercial cultivation. The genotypes were characterized using 30 SSR markers. Out of the 31 genotypes four each of drought tolerant (C306, HW2004, HD2888 and NI5439) and drought sensitive (HD2877, HD2012, HD2851and MACS2496) were used to study genotypic variation for root architecture at seedling stage after 6 and 9 days of sowing in petri dishes. One each of these contrasting highly drought tolerant (HW2004) and sensitive (HD2877) genotype was also observed for root growth and distribution at vegetative and reproductive stage under moisture stress conditions. All the 31 wheat genotypes were also grown in PVC pipes to study root traits at reproductive stage and in field for morpho-agronomical traits under moisture stress conditions.

## Experiments Conducted

### DNA Extraction and PCR Amplification

Genomic DNA was isolated from young green leaves using CTAB method with minor modifications [25]. Purified DNA was checked for quality and quantity using agarose gel electrophoresis as well as NanoDrop 8000 spectrophotometer (Thermo Scientific, www.nanodrop.com). Finally, diluted DNA was used to amplify SSR markers using eppendorf thermo-cycler (www.eppendorf.com). The PCR reaction was set in 10μl volume containing 1ŋg of genomic DNA, 5pmole each of forward and reverse primers, 0.1mM dNTPs, 1x PCR buffer (10mM Tris, pH 8.0, 50mM KCl and 50mM ammonium sulphate), 1.8mM MgCl_2_, and 0.3 unit of Taq DNA polymerase. The cycling conditions involved initial denaturation at 94°C for 3min, followed by 35 cycles of denaturation at 94°C, primer annealing at 50–60°C, and primer extension at 72°C each for 30seconds. A final extension at 72°C for 5min was done and the PCR products were stored at 4°C until electrophoresis. The PCR products were resolved by electrophoresis in 3.5% MetaPhor^™^ (Lonza) agarose gels in 1x TBE buffer and visualized by ethidium bromide staining. Gel photographs were documented using Syngene geldoc system (www.syngene.com). The polymorphism survey was carried out among genotypes using 30 SSR markers from Wheat Microsatellite Consortium (WMC) and Gatersleben Wheat Microsatellites (GWM) [26]. Primer sequences were obtained from Graingenes 2.0 database (http://graingenes.org).

### Molecular Analysis

The SSR profiles were scored based on the size of fragments amplified across all 31 wheat genotypes. The major allele frequency, polymorphism information content and genetic distance based clustering was performed with Unweighted Pair Group Method for Arithmetic average (UPGMA) tree using Power Marker v3.25 [27], and the dendrogram was constructed using MEGA 4.0 software [28]. Software GenAlEx V6.5 [29] was used for the Principle Coordinate Analysis and Analysis of Molecular Variance. The population structure was inferred using STRUCTURE 2.3.4 [30]. For Evanno plot, the Structure outputs were visualized using Structure Harvester [31]. In Structure, we assumed an admixed model with independent allele frequency and a uniform prior probability of the number of populations, *K*. All the runs with 50,000 MCMC replicates after a burn-in of 50,000 replicates were conducted for *K* = 1 to 10. Five independent runs were done for each value of *K* to generate our estimate of the true number of sub populations. The relation between genetic similarity identified by SSR markers and taxonomic distance measured by mean genetic distance were analysed using Jaccard’s similarity index and average taxonomic distance calculated by NTSYS-pc v2.1 [32].

### Root Traits Characterization at Seedling and Reproductive Stage

Single seed of each genotype, four drought tolerant (C306, HW2004, NI5439 and HD2888) and four drought susceptible (HD2851, HD2012, MACS2496 and HD2877) in a set of three replications were sown on filter paper segment in plastic petri dishes of 90mm diameter and 15mm height providing enough moisture to ensure germination. Initially, petri dishes were covered with cap for 3 days to retain moisture and after ensuring seed germination left opened at room temperature. Each seedling was given 2.0ml of water after three days. The seedlings were scanned using WinRhizo Tron MF software which is an image analysis system specially designed for root measurement (Regent Instruments Inc., Quebec, Canada). The observations were recorded on wheat seedlings at the interval of 6 and 9 days after sowing. The WinRhizo software gave root morphological data of total root length (mm), total projected area (mm^2^), total root surface area (mm^2^), total root volume (mm^3^), longest root (mm), coleoptile length (mm), and shoot length (mm).

The drought tolerant HW2004 and sensitive HD2877 genotypes were grown in field under drought stress conditions to study growth and distribution of roots. The seeds were sown under the soil in which a clear transparent pipe was inserted at an angle of 45 degree. The ambient moisture (24.6%) was provided to ensure seed germination. Root growth and development in the field under drought stress condition was monitored at vegetative and reproductive stages by taking digital images of roots with a WinRhizo root scanner having digital camera attached to a portable steel frame mounted in front of the chamber. The soil moisture was recorded 22.3% (0–15cm soil depth) and 24.5% (15–30cm soil depth) at vegetative stage and 17.3% at 0–15cm soil depth and 21.2% at 15–30cmsoil depth at reproductive stage.

Single wheat plants were grown in 200cm long and 10cm in diameter polyvinyl chloride (PVC) pipes for phenotypic evaluation in two successive years. Each pipe was loaded with thoroughly mixed soil composed of three parts soil from wheat field and one part vermi-compost. The bottom of each PVC pipe was covered with a cloth to intact soil in pipe. Appropriate soil moisture (24.2%) was maintained to ensure seed germination. Three seeds were sown directly in each pipe and only one healthy seedling was retained at 20 days after sowing. The root system and its components were measured at maturity under moisture stress condition. The soil moisture at maturity stage was recorded to be 8.3% (0-15cm soil depth) and 12.7% (15-30cm soil depth). The shoot of each plant was separated by cutting at the base of the stem. At maturity, wheat root system is densely entangled, difficult to separate individual roots and measure root numbers. To retrieve intact root systems, each PVC Pipe was laid in a tub fully filled with water for 16hrs of dimensions 4x2x1m of length x width x height, respectively. PVC pipe was pulled out gently without damaging the roots and leaving the sand core dissolved in water. The intact root system was floated to the water surface and washed carefully by hand to remove attached sand without damaging the root system. The traits like maximum root length in cm and root biomass in grams were recorded. Similarly, shoot traits like stem length, stem biomass, number of total tillers per plant and effective tillers per plant were determined for each genotype. Roots and shoots including leaves were dried in a forced-air drier for 24h at 80°C. Total root and shoot dry weight of each genotype was calculated. The root-shoot ratio was calculated as the ratio of the weight of the roots to the weight of the top of a plant. Similarly, root-shoot ratio for maximum root length and shoot length was also calculated.

### Phenotypic Traits Characterization in Field

One hundred and fifty eight wheat genotypes were evaluated for phenotypic characterization in Augmented Block Design under irrigated and drought stress conditions in field at New Delhi. The genotypes were planted in two rows of 1.5m each, 30cm apart in two replications at normal seed rate (100kg/ha). Five irrigations were provided under irrigated conditions while under drought stress condition only pre-sown irrigation was provided to ensure germination and then no irrigation was provided from sowing till maturity. Data were recorded on various morpho-agronomical traits under rainfed and irrigated conditions on three plants per row viz., plant height, peduncle length, spike length, spikelet number per spike, number of tillers per meter, thousand kernel weight (TKW in g), grain yield per meter and biological yield per meter. Plant height was measured from the tip of first spike on top of the plant to the base of the ground, spike length was measured excluding awns and peduncle length was measured from first node from the top of the plant to second node of the plant using a measuring scale (in cm). Total numbers of tillers per meter were counted for each genotype. Grain yield (in g) was recorded by weighing the grains obtained after threshing from one meter line of each genotype. Biological yield was calculated from the above ground biomass including the number of tillers per plant and number of grains per spike. Harvest index was calculated by measuring grain yield per biological yield. Thousand kernel weight was recorded by weighing thousand grains (in g) using electronic weighing machine.

### Statistical Analysis

The analysis of variance (ANOVA) of seedling traits for standard error (S.E.), critical difference (CD) and coefficient of variation (CV) was performed using OP Stat software. The coefficient of correlation among root and shoot traits at maturity was calculated using SPSS ver.19 software. Root traits and agronomical traits were used to form clusters based on their similarity index. The similarity matrices were used to construct a dendrogram for all the genotypes using NTSYS-pc based on UPGMA [32].

## Results

### Screening of Genotypes for Molecular and Morpho-Agronomical Characterization

A set of 158 genotypes (Australia, 72 and India, 86) were grown in field under irrigated and drought stress conditions during 2010–11 and 2011–12 (S1 Table). The significant variation among genotypes were recorded for plant height, number of tillers per meter, peduncle length, spike length, spikelet number per spike, thousand kernel weight, grain yield per meter, biological yield per meter and harvest index. The relative reduction in grain yield under drought stress condition is one of the trait which distinguish drought tolerant genotype with sensitive one. The genotypes were distributed in four coordinates on the basis of per cent reduction in grain yield with plant height under drought stress conditions. A total of 31 Indian origin wheat genotypes were selected on the basis of relationship of grain yield under drought stress conditions. Four genotypes from each coordinate I, II and IV while three genotypes from coordinate III were used for molecular and root traits characterization in the study (S1 Fig and S3 Table).

### Molecular Characterization Using SSR Markers

A total of 96 SSR markers were pre-screened in drought tolerant viz., C306, HW2004, NI5439 and HD2888 and drought sensitive, namely, HD2851, HD2877, MACS2496 and HD2012genotypes. Thirty of these SSR markers distributed over the chromosomes were selected for analyzing the genetic diversity in 31 genotypes (Table 1). The SSR markers showed a good amount of genetic diversity amplifying 103 alleles ranging from 2 to 6 with mean of 3.43 alleles per loci. However, the PIC analysis of SSR genotyping indicated biased distribution of highly polymorphic markers. The major allele frequency at the SSR loci varied between 0.32 (Xgwm437) and 0.94 (Xgwm124) with a mean value of 0.54. The allelic frequency and diversity determined by PIC values had an average value of 0.51 per SSR marker across all the genotypes. The range of PIC value was 0.11 in Xgwm124 to 0.74 in Xgwm437. The gene diversity for SSRs ranged from 0.12 (Xgwm124) to 0.78 (Xgwm437) with an average of 0.57 (Table 1). The genetic diversity index was usually positively correlated with a number of alleles and there was a positive linear relationship between the two indices within a given range. The coefficient of correlation indicated that the genetic diversity index was significantly and positively correlated with the number of alleles (r = 0.68, P<0.001). Genetic diversity (y) could be estimated by a curvilinear regression equation with independent variables of allele number per locus (x), i.e., y = 0.090 ln(x) +0.257 (1< x <7), R^2^ = 0.493(S2 Fig).

**Table 1:** Allele variation and PIC values for microsatellite loci (SSR) used for characterizing wheat genotypes.

Marker	Chromosome	Repeat Motif	Major Allele Freq.	Allele No	Gene Diversity	PIC
GWM122	2A	(CT)11(CA)31	0.581	3.000	0.570	0.504
GWM124	1B	(CT)27(GT)18	0.935	2.000	0.121	0.113
GWM148	2B	(CA)22	0.677	2.000	0.437	0.342
GWM413	1A	(GA)18	0.419	5.000	0.737	0.701
GWM311	6B	(GA)29	0.355	5.000	0.768	0.733
GWM332	7A	(GA)36	0.419	4.000	0.653	0.587
GWM369	3A	(CT)11(T)2(CT)21	0.548	3.000	0.595	0.528
GWM374	2B	(GT)17	0.548	2.000	0.495	0.373
GWM383	3D	(GT)27	0.548	2.000	0.495	0.373
GWM388	2B	(CT)4(CA)11(CA)12	0.645	2.000	0.458	0.353
GWM006	5A	(GA)40	0.452	5.000	0.656	0.594
GWM247	3B	(GA)24	0.355	4.000	0.693	0.633
GWM276	7A	(CT)24	0.516	6.000	0.639	0.587
GWM294	2A	(GA)9TA(GA)15	0.387	5.000	0.739	0.698
GWM428	1B	(GA)22	0.806	3.000	0.329	0.302
GWM437	7D	(CT)24	0.323	5.000	0.776	0.741
GWM448	2A	(GA)29	0.581	4.000	0.583	0.527
GWM499	5B	(GA)32	0.581	5.000	0.614	0.579
GWM501	2B	(CA)33	0.452	3.000	0.633	0.557
GWM539	2D	(GA)27	0.323	4.000	0.728	0.678
GWM570	6A	(CT)14(GT)18	0.452	3.000	0.641	0.567
GWM610	4A	(GA)17	0.742	3.000	0.408	0.362
WMC014	7D	(CT) (CA)	0.774	2.000	0.350	0.289
WMC048	4B	(GA)9	0.484	3.000	0.599	0.516
WMC089	4B	(CA)19	0.613	3.000	0.547	0.487
WMC093	1D	(GT)24	0.419	3.000	0.637	0.560
WMC388	3A	(GT)7	0.516	3.000	0.612	0.541
WMC429	1D	(GT)31	0.419	3.000	0.653	0.580
WMC457	4D	(CA)13	0.677	3.000	0.470	0.405
WMC479	7A	(GT)16	0.710	3.000	0.450	0.402
Mean			0.542	3.433	0.570	0.507

The utility of SSR markers was used to characterize and evaluate the genetic diversity at seven homoeologus groups of A, B and D genomes among 31 genotypes. The 30 SSR loci were well distributed among the A, B and D genomes wheat detecting 47, 34 and 22 alleles, respectively (Table 2). The average genetic richness was 3.92 for the A genome, 3.09 for Band 3.14 for D genome, and the genetic diversity index for the three genomes was in order of 0.60, 0.58 and 0.51for A, B and D genomes, respectively. ANOVA revealed that the A genome had higher magnitude of genetic diversity followed by D and the B genomes. Significant differences were observed between the B and D genomes (P = 0.01).

**Table 2:** Genetic diversity at the genome level of the 31 wheat genotypes revealed by 30 SSR markers.

Genome	No. of Loci	MAF (range)	Mean Allele Number (range)	Gene Diversity (range)	PIC (range)
A	47	0.52(0.38–0.74)	3.91(3–6)	0.60(0.44–0.73)	0.55(0.36–0.70)
B	34	0.58(0.35–0.93)	3.09(2–5)	0.51(0.12–0.76)	0.45(0.11–0.73)
D	22	0.49(0.32–0.77)	3.14(2–5)	0.58(0.47–0.77)	0.51(0.28–0.74)
**whole genome**	**103**	**0.53(0.32–0.93)**	**3.38(2–6)**	**0.57(0.12–0.77)**	**0.50(0.11–0.74)**

MAF = Major Allele Frequency; PIC = Polymorphism Information Content

The genetic diversity among the seven homoeologous groups of wheat revealed that the average genetic richness for homoeologous groups 5 (5.0), 6 (4.0) and 7 (4.0) was higher than that for groups 1 (3.1), 2 (3.0), 3 (3.0) and 4 (3.0). In addition, the average genetic diversity index for homoeologous groups 6 (0.70), 5 (0.63) 3 (0.59), 2 (0.58) and 7 (0.57) was higher than that for 4 (0.50) and 1 (0.49) (Table 3).

**Table 3:** Genetic diversity in 7 homoeologous groups of the 31 wheat genotypes as revealed by 30 SSR markers.

Homoeologous group	MAF (range)	Mean Allele number (range)	No. of alleles	Gene Diversity(range)	PIC (range)
1	0.60(0.41–0.93)	3.20(2–5)	16.00	0.50(0.12–0.73)	0.45(0.11–70)
2	0.52(0.32–0.67)	3.13(2–5)	25.00	0.58(0.43–0.72)	0.50(0.37–0.69)
3	0.49(0.35–0.54)	3.00(2–4)	12.00	0.60(0.49–0.69)	0.52(0.37–0.63)
4	0.63(0.48–0.74)	3.00	12.00	0.51(0.40–0.59)	0.44(0.36–0.51)
5	0.52(0.45–0.58)	5.00	10.00	0.63(0.61–0.65)	0.59(0.57–0.59)
6	0.40(0.35–0.45)	4.00(3–5)	8.00	0.70(0.64–0.76)	0.65(0.56–0.73)
7	0.55(0.32–0.77)	4.00(2–6)	20.00	0.57(0.34–0.77)	0.52(0.28–0.74)

**Fig 2:**
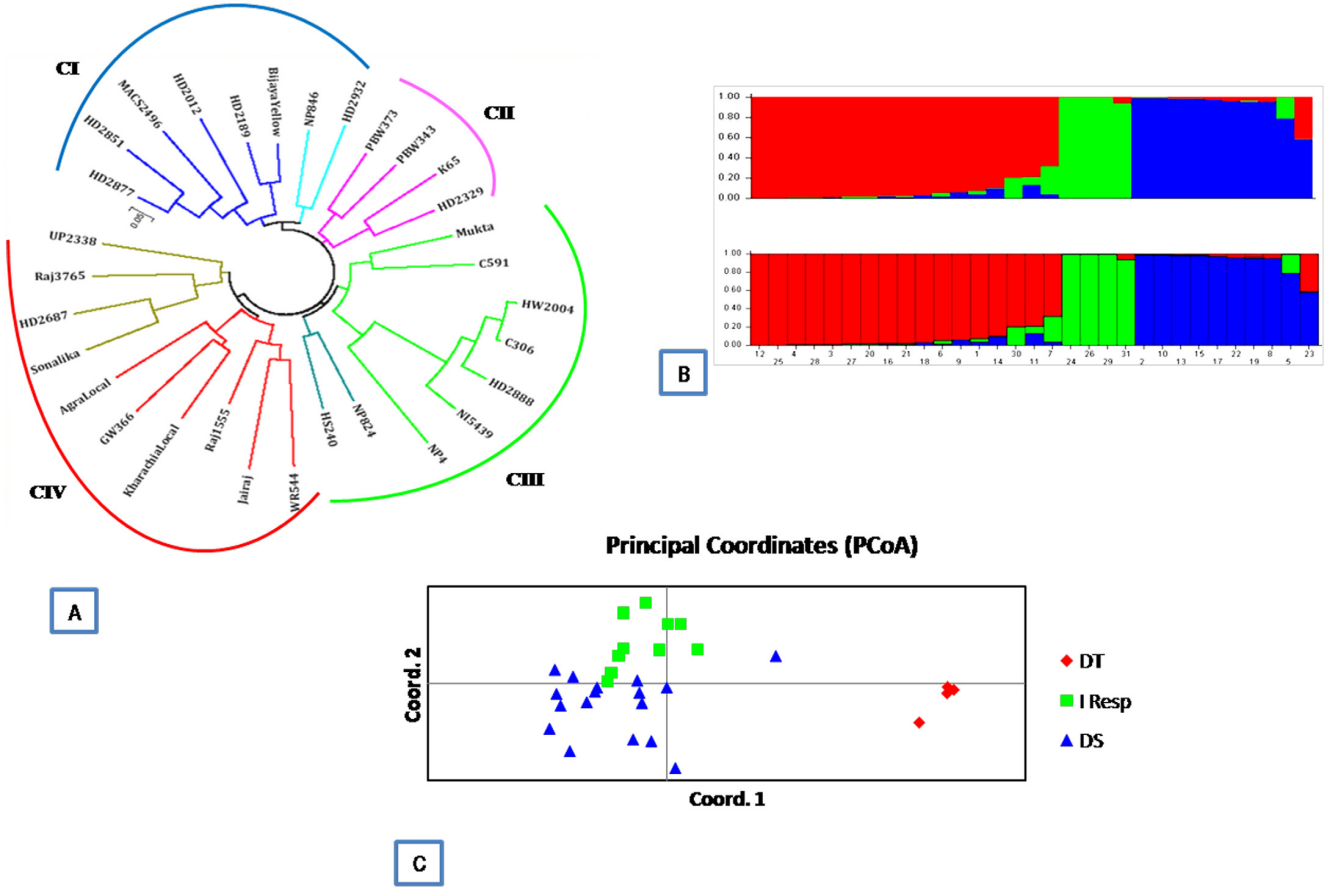
Categorization of 31 wheat genotypes revealed by 30 SSR markers. (A) UPGMA tree constructed using Jaccard’s similarity coefficient; (B) Three subgroups formed from STRUCTURE analysis, Model based population structure plot for each variety with K = 3. Color codes are as follows: Population I red, Population II green, population III blue; (C) Principal coordinates analysis The genotypes are grouped as DT = drought tolerant genotypes, Intermediate Responsive = moderate tolerant and sensitive genotypes and DS = drought sensitive genotypes.

Using Jaccard’s genetic distance method, a dendrogram (Fig 2A) was constructed from the genetic dissimilarity matrix, derived from 30 SSR markers. In the UPGMA tree, wheat genotypes were grouped into 4 main clusters which corresponded well with an indication towards drought stress as drought sensitive (DS), drought tolerant (DT), moderately tolerant (MT) and moderate sensitivity (MS) (S4 Table). The drought sensitive genotypes were clustered into I group having 8 genotypes viz., HD2877, HD2851, MACS 2496, HD2012, HD2189, HD 2932, Bijaga Yellow (durum) and NP846. Cluster II included 4 genotypes, namely, PBW343, PBW373, K65 and HD2329 with moderate sensitivity to drought stress. Cluster III comprised of 9 drought tolerant genotypes, namely, Mukta, C591, C306, HW2004, HD2888, NI5439, NP4, NP824 and HS240), while cluster IV was consisted of 10 moderately tolerant genotypes viz.,Raj1555, Jairaj (both durum), WR544, GW366, Kharachia Local, Agra Local, UP2338, Raj3765, HD2687 and Sonalika.

The 30 SSR markers were also used for estimation of population structure among the same set of 31 wheat genotypes using STRUCTURE V2.2.2 software. A total of 3 subpopulations (K) were identified based on maximum likelihood and delta K (ΔK) values. The Structure analysis categorized genotypes as pure or admixture; genotypes with more than 0.80 score were considered as pure and less than 0.80 as admixture. In SSR-based structure, population I consisted of 8 drought sensitive and 4 moderately sensitive genotypes along with 5 moderately tolerant genotypes, while population II composed of four highly drought tolerant genotypes. Population III consisted of 10 wheat genotypes with moderate tolerance to drought stress including two durum wheat genotypes Raj1555 and Jairaj (Fig 2B). According to SSR-based structure analysis GW366 and HD2687 were found admixed, while all the genotypes were found pure. All the 31 genotypes were clustered into three major groups inferring that the grouping of genotypes using STRUCTURE was according to UPGMA clustering which is based on genetic relationship to drought stress. The ancestry details for determination of population structure of 31 wheat genotypes are given in S3 Table. SSR-based principal coordinates analysis clustered the accessions into three major groups which closely agreed with the UPGMA-based tree and Structure grouping. The first three coordinate axes accounted for 33.23% of the variation observed (Fig 2C).

The first axis explained 15.09% of genetic variation followed 10.43% by second axis and 7.71% by the third axis. All of the sensitive and intermediate (moderate sensitive and tolerant) to drought were plotted in first half of the coordinates while highly drought tolerant genotypes were plotted in second lower half of the coordinates. It gives a clear representation of genotypes distributed in three distinct groups depending upon response to moisture stress. In addition, an analysis of molecular variance (AMOVA) procedure was used to estimate the partitioning of genetic variance among and within populations. According to SSR markers the percentages of genetic variation within population were 76% and among population was 24% (Table 4).

**Table 4:** AMOVA summary for SSR.

Source	df	SS	MS	Est. Var.	%
Among Pops	2	225.80	112.90	9.27	24%
Within Pops	28	833.55	29.77	29.77	76%
Total	30	1059.35	142.67	39.04	100%

### Genotypic Variability for Root Traits

**Fig 3:**
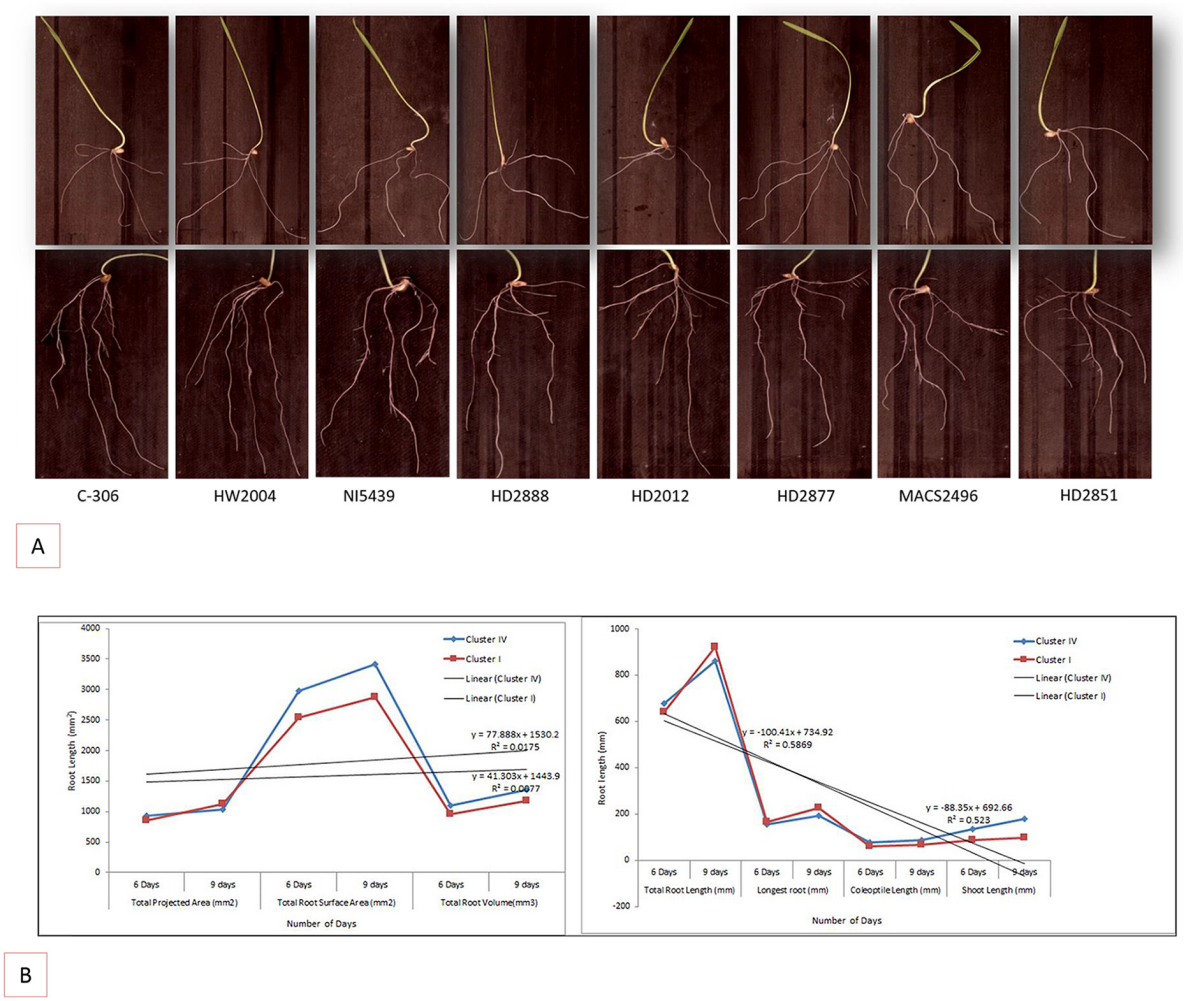
Root traits studied at seedling stage. (A) Root distribution pattern analyzed in drought tolerant and susceptible genotypes grown under well-watered conditions in petri dishes at the interval of 6 and 9 days. (B) Relationships between root traits measured at 6 and 9 days old seedling estimated from two Clusters (I and IV) of wheat genotypes a. Total root length, Longest root, Coleoptile length and Shoot length. Total Projected area, Total Surface area and Total root volume. The solid lines indicate the best fit regression line and 95% confidence length intervals, respectively.

The ANOVA was calculated on 6 and 9 days old seedling on 8 drought tolerant (Cluster IV) and drought sensitive genotypes (Cluster I) grouped based on Jaccard’s genetic dissimilarity matrix. The analysis of variance did not show any considerable variability between drought tolerant (C306, HW2004, NI5439 and HD2888) and drought sensitive (HD2851, MACS2496, HD2012 and HD2877) genotypes at 6 days old seedling stage indicating no significant effect on total root length, longest root, total projected area and total root volume. Although a very high and significant variation was recorded for total surface area, coleoptiles length and shoot length (S6 Table and Fig 3A). However, 9 days old seedlings showed critical difference in longest root, total projected area, total surface area, coleoptile length and shoot length and no difference in total root length and total root volume (Fig 3B). The drought tolerant genotypes had the longest coleoptile and shoot compared to drought sensitive genotypes.

**Fig 4:**
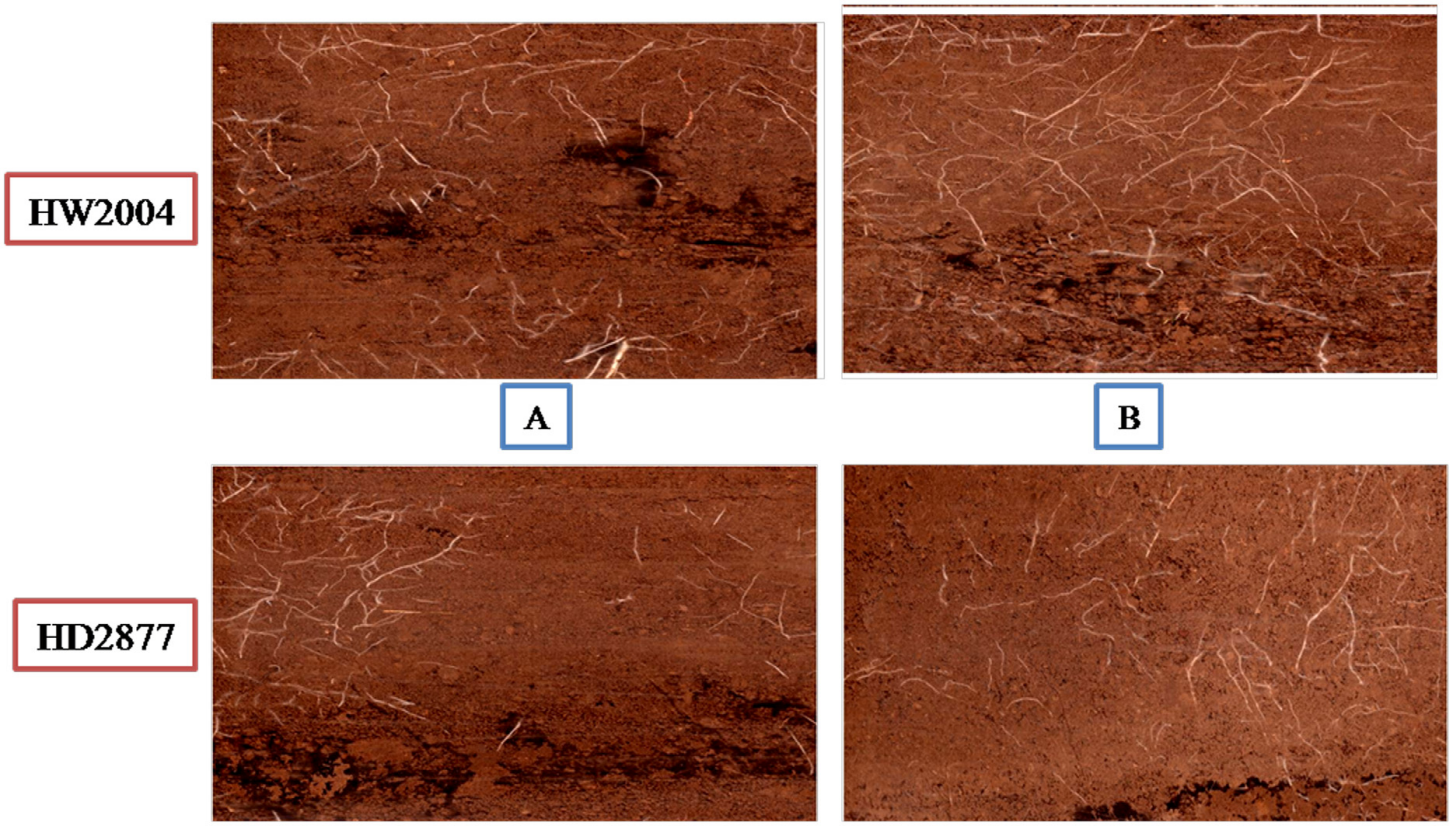
Digital images taken from field under drought stress conditions at vegetative and reproductive stages. Differences in the root distribution and root length production Drought tolerant wheat genotype HW2004 and drought sensitive genotype HD2877 at vegetative (A) and reproductive stage (B).

The data were also recorded on root growth and distribution in the vegetative and reproductive stages of drought tolerant HW2004 and drought sensitive HD2877 genotype in field under drought stress conditions. The difference between HW2004 and HD2877 illustrates, an example, in the uniformity of root distribution as traced on the digital images taken from WinRhizo root scanner (Fig 4A & 4B).

The visible evaluation of root images of drought tolerant genotype HW2004 indicated that the roots occupied the available soil volume in each section more uniformly, while HD2877 exhibited a less even root distribution. The significant genotypic variation in mean root length was not observed in the early growth stages but differed substantially at later growth stage. Although, HW2004 had characteristic feature of compact root system with less root growth laterally and more root length at depth while HD2877 have higher horizontal root spread at reproductive stage.

**Fig 5:**
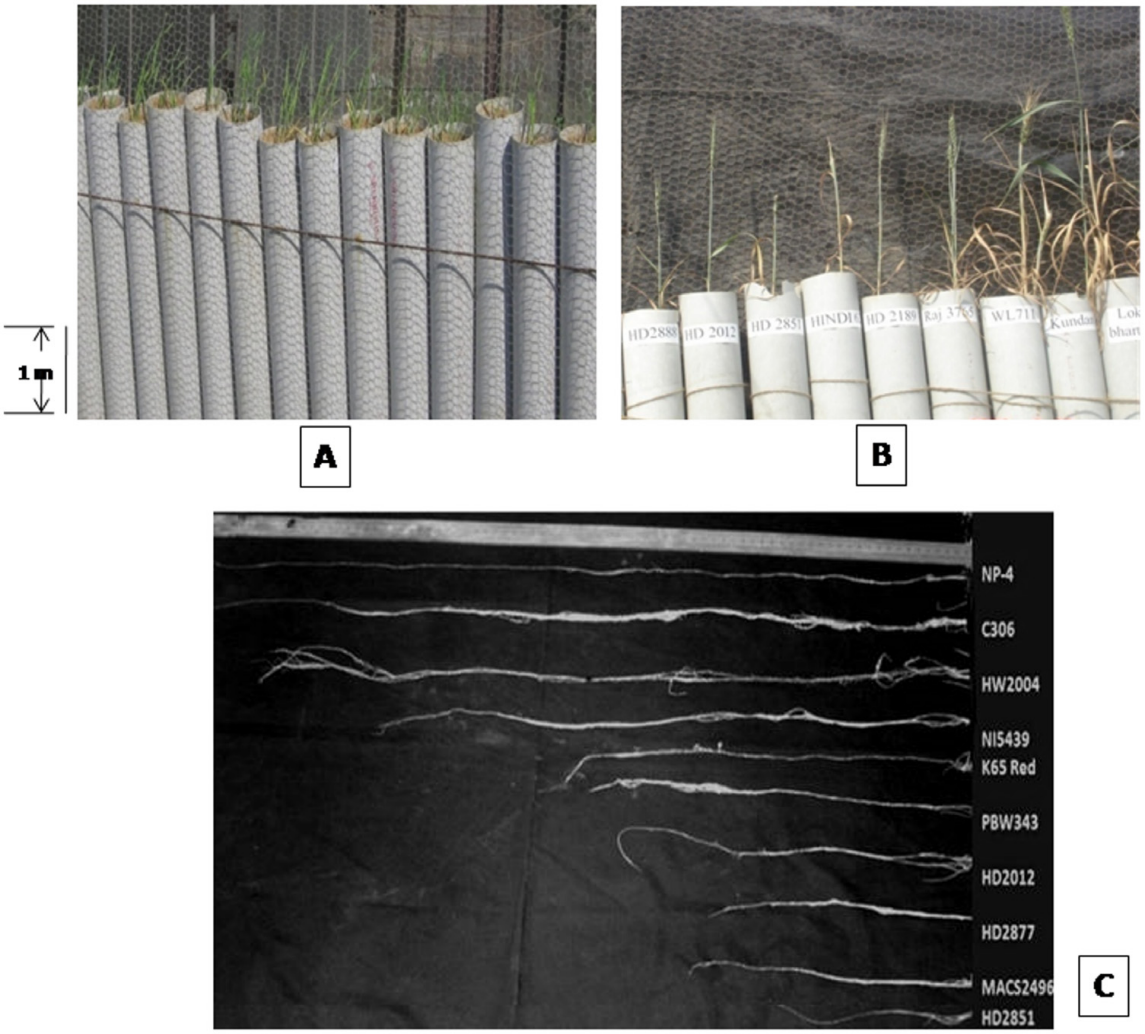
Root traits of 31 wheat genotypes studied under drought stress condition at reproductive stage. Wheat genotypes at seedling (A)and Maturity (B) stage grown in PVC pipes to examine root growth and development; (C) Representative picture showing variation in maximum root length of ten wheat genotypes grown in PVC pipes under drought stress conditions.

Considering 31 wheat genotypes, significant difference was observed among the root traits at maturity under moisture stress conditions in PVC pipes (Fig 5A & 5B). Drought tolerant genotypes grew to maximum depth as compared to sensitive ones. The root length of genotypes varied from 63cm (Kharachiya Local) to 137cm (C306) with a mean value of 94.85cm.The average root length of 120cm was recorded for those genotypes which have been released for rain-fed conditions. The root biomass ranged from 0.47g (HD2189) to 1.91g (C306) with an average value of 0.58 (S5 Table).

Based on the data on the root traits, two separate clusters were formed using NJ’s Jaccard similarity coefficient (S3A Fig). Group II included genotypes (Bijaya Yellow, C306, C591, HW2004, HD2888, NI5439, NP4, NP824 and NP846) released for rain-fed or moisture deficit conditions. Another wheat variety, WR544 (Pusa Gold), released for late sown or very late sown conditions possessing characteristics for thermo-tolerance and drought tolerance was also found in the same group. Group I was divided into sub groups, Group IA and Group IB. Group IA included genotypes released for timely sown and irrigated conditions with an exception of HS240 which is rainfed variety released for Hill zone. Jairaj (T. *durum*) was also included in this cluster. While Group IB included genotypes like Agra Local, UP2338 and K65, highly sensitive to drought stress with an exception to Mukta, drought tolerant bread wheat and Raj1555, durum wheat variety.

### Phenotypic Traits Analysis

The plant height and peduncle length of the genotypes grown under rain-fed conditions in field showed a great range of variability with the range of 70cm (HD2851) to 125cm (C306 and NP824) with an average value of 83.31 for plant height and 26cm (HD2877 and PBW343) to 45cm (HW2004 and NI5439) with an average value of 27.85 for peduncle length, respectively. The genotypes showed a wide range of diversity under moisture stress condition, as grain yield per plant had an average value of 2.39 with a range of 2g (HD2687) to 5.71g (C306), biological yield per plant in the range of 12.21g (HD2851) to 19.61g (C306) and thousand kernel weight in the range of 21g (HD2851) to 42g (C306 and HW2004) (S5 Table). Observation recorded on agro-morphological traits under moisture stress conditions in the field also classified all the genotypes in two groups. In this classification, genotypes sensitive to drought stress with relatively low yield were grouped in Group I. Group I was further sub divided into Group IA viz., Agra Local, HD2012, HD2851, MACS2496 and HD2877 and Group IB viz., GW366, HS240, Kharachiya Local, HD2189, Raj3765, Sonalika, UP2338, HD2329, PBW343, PBW373, WR544, HD2932, HD2687, Jairaj and Raj1555 depending on yield and yield contributing traits under water deficit conditions. The drought tolerant genotypes viz. HD2888, NP824, C306, HW2004, Mukta, NP4, NP846, C591, NI5439, K65 and Bijaya Yellow with high yield potential under drought stress conditions were placed in Group II (S3B Fig).

Significant and positive coefficient of correlation was recorded among genotypes for morphological and agronomical traits under moistures stress conditions in field and root traits in PVC pipes. Maximum root depth (MRD) showed a positive correlation at 0.01 level with shoot height of plant grown in PVC pipes, shoot and root length ratio, root mass, root and shoot mass ratio, plant height of genotypes grown in field, peduncle length, tiller number per plant, biological yield per plant, grain yield per plant and thousand kernel weight (S7 Table). Plant height (PH) showed positive and significant correlation at 0.01 level with SH, RL, RL: SH, SM, TN PL, BYD, GYD, TKW. Grain yield per plant also showed a strong and positive correlation with shoot height, root length, biological yield per plant, thousand kernel weight and number of tillers per plant.

### Relationship between Genetic Similarity and Grain Yield

**Fig 6:**
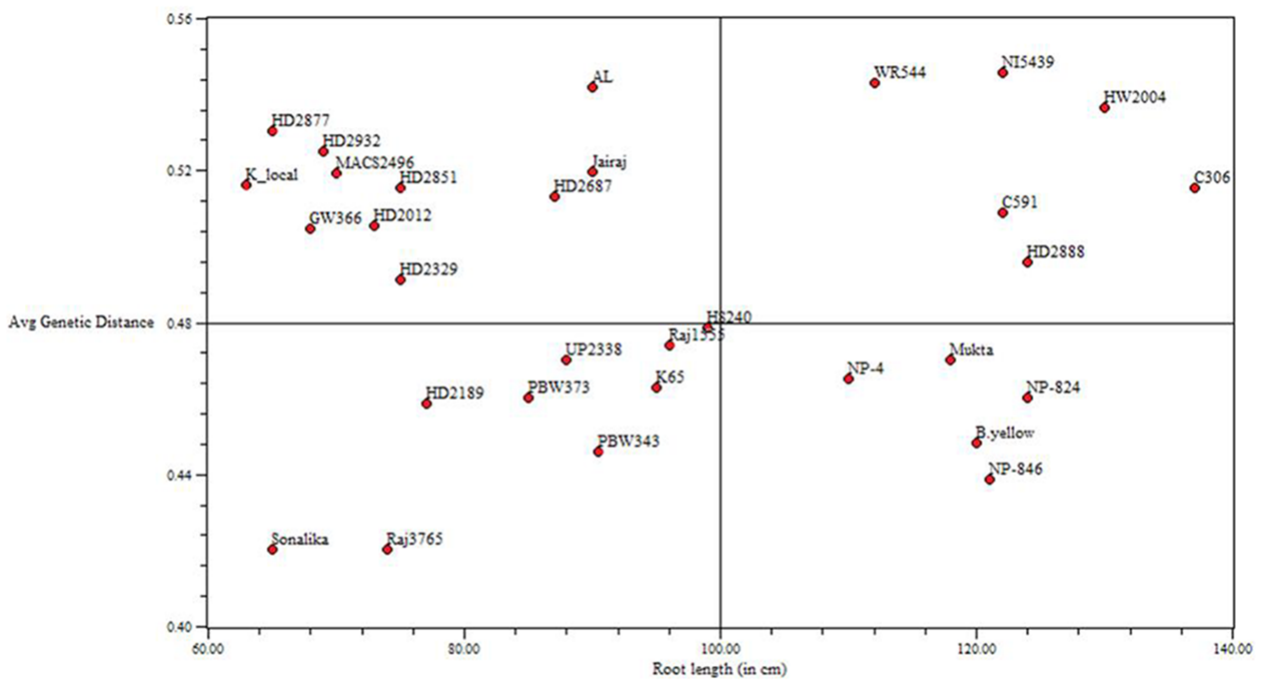
Correlation between genetic similarity index and average taxonomic distance for root length per plant. The genotype pair and average taxonomic distance are represented by symbol (0).

The relationships between Jaccard similarity index based on simple sequence repeat markers and average taxonomic distance, based on root length were analyzed by making comparisons between the two matrices (Fig 6). The correlation between genetic similarity and the taxonomic distance was found highly significant (*r* = −0.*50*;*P* = 0.005).

## Discussion

The conservation and efficient use of available resources in the form of germplasm requires proper understanding of genetic diversity. To evaluate genetic diversity in wheat several tools have been widely used like study of phenotypic traits [33] and molecular markers [34]. The present study, combined analysis of information based on phenotypic traits (root and shoot traits for morphology and agronomy) under drought stress conditions with molecular markers was done. Phenotypic traits of genotypes are largely affected by environment interaction while molecular markers are irrespective to such kind and estimate the effect at neutrality.

### Molecular Characterization Using SSR Markers

This investigation was carried out to find out the allelic diversity existing among a collection of 31 wheat genotypes using 30 SSR markers. The estimated genetic diversity among the studied genotypes was with mean value of 0.57 and 3.43 alleles per locus. However, previous studies reported number of alleles per locus in the range of 4.81 to 18.1 and mean genetic diversity values ranging from 0.46 to 0.77 in wheat [35, 36, 37, 38]. The differences in results obtained by different researchers are reflected due to the type of material (landraces, wild species etc.,) used in the study.

The three genomes showed significant differences in largeness of genetic diversity. Genome A showed the highest diversity (0.60) followed by D (0.58) and B (0.52) genomes. These results are in contrary to the previous reports, which showed genetic richness from highest to lowest in order of B>A> D and genetic diversity indexes were in the sequence of B>D>A [39,37,7]. Also, Liu [40] using RFLP markers reported higher genetic differentiation on B genome than on the A genome between *Triticum aestivum* and *T*. *spelta*. Chao [41] revealed lower levels of polymorphism in the D genome than in the A and B genomes indicating that lateral gene flow from tetraploid to hexaploid species was frequent, while it was very limited between the diploid *Ae*. *tauschii* and hexaploid wheat. We confer that there is a lower level genetic diversity in collected wheat genotypes, especially for the B genome. Consequently, inclusion of more wheat genotypes from other resources has the potential to enhance the genetic diversity of breeding materials.

The dendrogram generated from similarity coefficients indicated four main groups (Fig 2A) tested for statistical significance at the 0.01 level of probability, respectively. The Cluster I (CI) included drought sensitive genotypes, Cluster II (CII), had moderately drought sensitive genotypes and Cluster III (CIII) was relatively homogeneous consisting well known drought tolerant genotypes. The clustering of genotypes proved the suitability of SSRs in detecting alleles characteristic of cultivars from distant genetic background. The closer association of C306, with the drought tolerant wheat HW2004, HD2888 (C306/*T*. *sphaerococcum*//HW2004) and NI5439 to a large extent reflects selection for genomic regions present in the drought tolerant genotypes. Cluster IV (CIV) included genotypes which showed intermediate response i.e. moderately tolerant to drought stress.

The tree generated by SSR markers is in agreement with the STRUCTURE results. In SSR-based structure, population I consisted 17 genotypes including moderate sensitive to moderate drought tolerant genotypes like Mukta, C591, NP4, NP824 and HS240. Because these genotypes possess similar characteristic features such as height, long peduncle, long coleoptiles and higher biomass responding to moisture stress conditions. NP4, C591 and NP824 were released in India well before the green revolution took place and the irrigation system was not well developed. Although, based on molecular analysis, these genotypes with moderate tolerance formed a separate cluster (CIII) along with the highly drought tolerant genotypes. Population II composed of four highly drought tolerant genotypes, C306, HW2004, HD2888 and NI5439 (Fig 2B). Population III consisted of 10 wheat genotypes with intermediate response to moisture stress including two durum wheat genotypes Raj1555 and Jairaj. According to SSR-based population structure analysis GW366 and HD2687 were found admixed, while all the genotypes were found pure. Inferred ancestry details for determination of population structure of 31 wheat genotypes are given in S4 Table. The genetic variation obtained by SSR markers has been explained by first axis of PC analysis (Fig 2C). SSR markers gave better results in grouping of the current set of genotypes according to genetic constitution having tolerance towards drought stress. Highly drought tolerant group of four genotypes was found distinct with the highly sensitive and intermediate groups. All the three methods (UPGMA-tree, PCoA and Structure) used in the current study to assess wheat genotypes in response to drought stress, were found meaningful as most of the groups were co-linear in all the grouping methods as has been advocated earlier by [37].

### Phenotypic Traits Characterization

The requisite of plants grown under water deficit conditions lead to changes like increase in depth, width and branching of root systems resulting into reduce water stress. The information of plant genetic background for adaptability under adverse environment is essential for effective breeding. However, due to limited information is available regarding heritability and inheritance characters of wheat root system but infers that traits are governed by polygenetic system [42]. The primary objective of the present study was to characterize the differences in the patterns of root growth and development in wheat genotypes differing in tolerance to water limitation. The results obtained from the petri dishes on root growth and development of drought tolerant (HW2004, C306, HD2888 and NI5439) and the drought sensitive (HD2877, HD2851, HD2012 and MACS2496) genotypes were compared. This system allowed for continuous nondestructive measurements of root length visible at 6 and 9 days old seedling stage. The genotypic differences in respect of potential root growth under continuously water supply in the absence of any physical or chemical constraints were visible. The wheat genotypes of clusters drought tolerant and drought sensitive (obtained from SSR marker characterization Fig 2A) at seedling stage of 6 and 9 days did not show any significant variation in root traits except longer coleoptile and shoot and highest absorptive surface area in drought tolerant genotypes [43]. However at 9 days stage several fibrous root and root hairs were visually seen in all the genotypes with drought tolerant genotypes having longer total root compared to drought sensitive genotypes.

Root architecture differed markedly in the drought tolerant, HW2004 from drought sensitive genotype, HD2877. HW2004 exhibited the most compact root architecture and relatively greater lateral root spread indicating that the drought tolerant genotypes may have advantage over HD2877, a sensitive genotype with horizontal seminal root growth and superficial root systems due to their different genotypic constitution. The development of an extensive root network at depth in response to soil drying in surface layers has been reported as an important drought-adaptive root trait in many crop species [44] including wheat [45,46]. The number of roots and their distribution pattern plays an adaptive role in drought stress conditions [47].

The 31 wheat genotypes were also grown in PVC pipes to allow plant growth and development until maturity in stored soil moisture. The drought tolerant genotypes had an average root length of 120cm while maximum root length (137cm) was recorded for C306, a well known and tested drought tolerant genotype. The genotypes suitable for high input with ensured irrigation produced shorter roots in the range of 63 to 90cm. This may be largely due to the genetic constitution of the genotypes, environment and G x E interaction. Additive genetic systems influence the root systems which ensure root quantity and depth under adverse conditions [48]. The continued growth of roots of drought tolerant genotypes under dry soil is important to avoid drought [49]. Maximum root depth is potentially a good selection criteria for drought tolerance as it has significant and positive correlation with plant traits grown in PVC pipes like shoot height, shoot/root length ratio, root mass, root and shoot mass ratio and plant traits grown in field such as plant height, peduncle length, tiller number per plant, biological yield per plant, grain yield per plant and thousand kernel weight. Previous studies reported root length has positive correlation with root shoot ratio [50]. Plants with longer root possess higher biomass and surface area in comparison to small and shallow rooted plants. Rebetzke and Richards [51] and Liao *et al*. [52], found that improved water use efficiency and grain yield of crops, which depend on seasonal rainfall, can be achieved by root traits like higher root dry matter, root length density and root surface area leading to increased early vigour and pre-anthesis water use. In the present study, genotypes known for drought tolerance had an average root biomass in the range of 1.40 to 2.21g while susceptible genotypes produced 0.46 to 0.70g. The water and nutrient absorption is increased with the higher root biomass leading to increased yield [53]. The root biomass is also positively correlated with grain yield under moisture deficit conditions [54]. Most of the genotypes suitable for drought stress conditions are tall ranging from 107cm to 125cm as compared to drought sensitive, which are dwarf (60cm to 95.8cm) (S5 Table). Tall genotypes possess longer root with the capacity to extract water from the deeper soils and also translocate the stored materials in the stem leading to higher grain yield under drought conditions in comparison with genotypes having shorter height [55, 56]. A wide range (26-45cm) for peduncle length was observed among all the genotypes. The drought tolerant genotypes had comparatively longer peduncle (42.0 to 45.0cm) to drought susceptible genotypes (26–33.0cm). HD2888 among the tolerant genotypes was an exception (33.0cm) because *T*. *sphaerococcum* involved in its pedigree produces short peduncle. In the present study, peduncle length exhibited positive correlation with plant height in field, shoot height in PVC pipe, root length, root shoot ratio, root biomass and tiller number under moisture stress conditions. The higher peduncle length in drought tolerant genotypes may be due to higher plant height. The peduncle plays an important role in grain filling because it has higher green area which is likely to contribute to photosynthesis leading to increased grain yield under moisture stress [57, 58].

Under the influence of drought stress plants alter their growth morphology. Above ground mass is reduced while below ground mass is increased [59]. Wheat genotypes, namely, Bijaga Yellow, C591, C306, HW2004, NP846, NP824 HD2888, K65, Mukta displayed root shoot ratio ≤3.0, an adaptive feature under drought stress. The genotypes vary in capability of assimilate partitioning between roots and shoots [60]. The phenotypic variability for root: shoot ratio is controlled by additive gene effects [61]. The genotypes with good water management produce high yields under water deficit conditions [62]. Thousand kernel weight (TKW) produced by drought tolerant genotypes was higher (37.0–42.0g) than the drought sensitive genotypes (21–24.6g). It is generally observed that TKW has positive and direct effect on grain yield under water stress conditions as evidenced by HW2004 producing maximum TKW, whereas MACS2496 had lowest. The decrease in TKW and weight of kernels per spike under water deficit is leading to reduced grain yield [63, 64]. TKW also showed significant positive correlation with root depth, root biomass, and shoot height of plants grown in PVC pipes and under moisture stress field conditions with plant height, biological yield per plant and grain yield per plant. The drought tolerant genotypes comparatively gave more grain yield/plant (4.8–5.5g) than the sensitive genotypes (2.2–3.3g). Apart from other yield contributing traits, number of tillers per plant plays a major role in determining the grain yield under moisture stress conditions. C306 produced highest number of tillers per plant, a most ideal rain-fed genotype released in 1965 still yields higher than other released cultivars for same ecological conditions. The number of tillers and plant height enhance biological yield with minimum (12.2g) and maximum (18.56g) dry matter was recorded in HD2851 and C306. It is evident that moisture stress decreased the biological yield (BYD) in drought susceptible genotypes. Biomass (total dry matter) production is an important criterion to judge drought tolerance in crop breeding [65]. In all the thirty one genotypes grain yield showed positive and significant correlation with traits of plants grown in both PVC pipes like shoot height, maximum root depth, root shoot ratio and in field like plant height, peduncle length, number of tillers per plant, thousand kernel weight and biological yield per plant. Positive and significant associations between yield and other traits mentioned above have also been reported in other cereals [66, 67]. The traits showing strong correlations with yield should be given more emphasis in breeding to improve yield in wheat under moisture stress conditions. Clustering of genotypes on the basis of morpho-agronomical traits and root architecture traits are according to grouping based on SSR markers analysis. Highly significant (*P* = 0.005) correlation was observed between genetic similarity index based on SSR markers and taxonomical distance based on root length (Fig 6). This indicated that genotypes with closer genetic relationships will be similar at least for root length related traits. However, there is a fundamental difference in the concepts underlying both the measures of genetic diversity. The morphological data is an indirect measure of genetic diversity, which qualifies the degree to which two genotypes are identical by morphology. In contrast, the rationale for using genetic similarity estimates based on molecular data is that the proportion of amplified products shared between two genotypes is an indicator of their resemblance in the DNA sequence across the entire genome [68].

## Conclusion

This study in wheat correlates study of root traits at 6 and 9 days old seedling in control condition with root growth and distribution pattern at vegetative and reproductive stage under drought stress conditions in field. The root and shoot traits of genotypes grown in PVC pipes were further correlated with morphological and agronomical traits grown under drought stress condition. The study confirms that under control conditions genotypes at seedling stage of 6 and 9 days did not show much significant variation in root traits except longer coleoptiles and shoot and higher absorptive surface area for drought tolerant genotypes. But, at the vegetative and reproductive stage drought tolerant genotype produce a very compact and high depth root compared to drought sensitive genotype under drought stress conditions. The SSR marker analysis showed great diversity range among the genotypes with A genome having the highest value compared to B and D genome. This is also the new finding which suggest A genome is more genetically rich. The SSR marker based analysis using UPGMA tree, Population structure and principal coordinate in the current study assess wheat genotypes in response to drought stress, were found meaningful as most of the groups were co-linear in all the grouping methods. NJ tree constructed using agronomical and root traits also give same clustering pattern as shown by genotypic variation. This study will provide a platform to identify genotypes using SSR markers on the basis of root and shoot traits under drought stress conditions.

## Supporting Information

S1 Fig Correlation between average plant height (PH) and grain yield per plant (GYD). The genotype pair and average taxonomic distance are represented by symbol (0). The four different coordinates are I, II, III and IV. Blue circle indicate genotypes selected for molecular and root characterization.
(TIF)

S2 Fig Scatter plot of gene diversity vs. number of alleles per locus. (TIF)

S3 Fig Dendrogram of 31 genotypes of wheat clustered in respect of similarity estimates based on (A) agronomical traits in field and (B) root traits in PVC pipes under moisture stress conditions. (TIF)

S1 Table List of wheat genotypes grown in field under irrigated and drought stress condition. (XLSX)

S2 Table Weekly average of maximum and minimum temperature, rainfall, relative humidity, evaporation rate and wind speed) for wheat crop growing season during 2010–11 and 2011–12 at New Delhi. (XLSX)

S3 Table List of wheat genotypes used in the root architecture study. (DOCX)

S4 Table Genotypes in various clusters based on 30 simple sequence repeat markers analysis. (DOCX)

S5 Table Observations recorded for root traits in PVC pipes and morpho-agronomical traits in field under drought stress condition. (XLSX)

S6 Table Variation recorded among drought tolerant and drought sensitive genotypes for various root traits at 6 and 9 days old seedling stage under control conditions. (XLSX)

S7 Table Correlation among the different root and shoot traits analyzed in 31 genotypes of wheat grown in the PVC Pipes and field under drought stress condition at New Delhi. (DOCX)
