# Supplementary figures and images for: Molecular and Morpho-Agronomical Characterization of Root Architecture at Seedling and Reproductive Stages for Drought Tolerance in Wheat
Source: PLoS One. 2016 Jun 9;11(6):e0156528. doi: 10.1371/journal.pone.0156528 (PMC4900657; doi:10.1371/journal.pone.0156528)

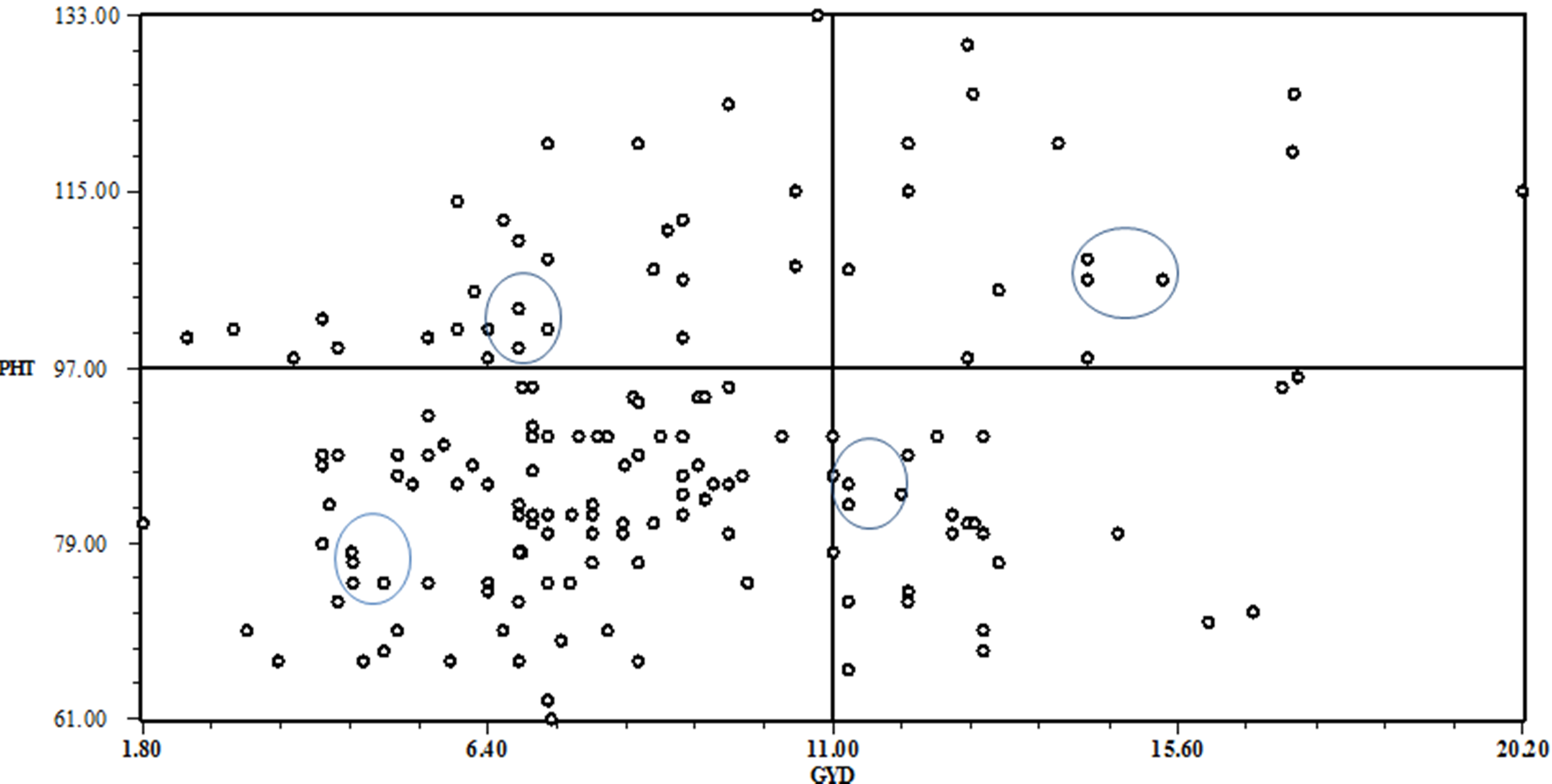

Supplement: S1 Fig — The genotype pair and average taxonomic distance are represented by symbol (0). The four different coordinates are I, II, III and IV. Blue circle indicate genotypes selected for molecular and root characterization. (TIF) [file pone.0156528.s001.tif]

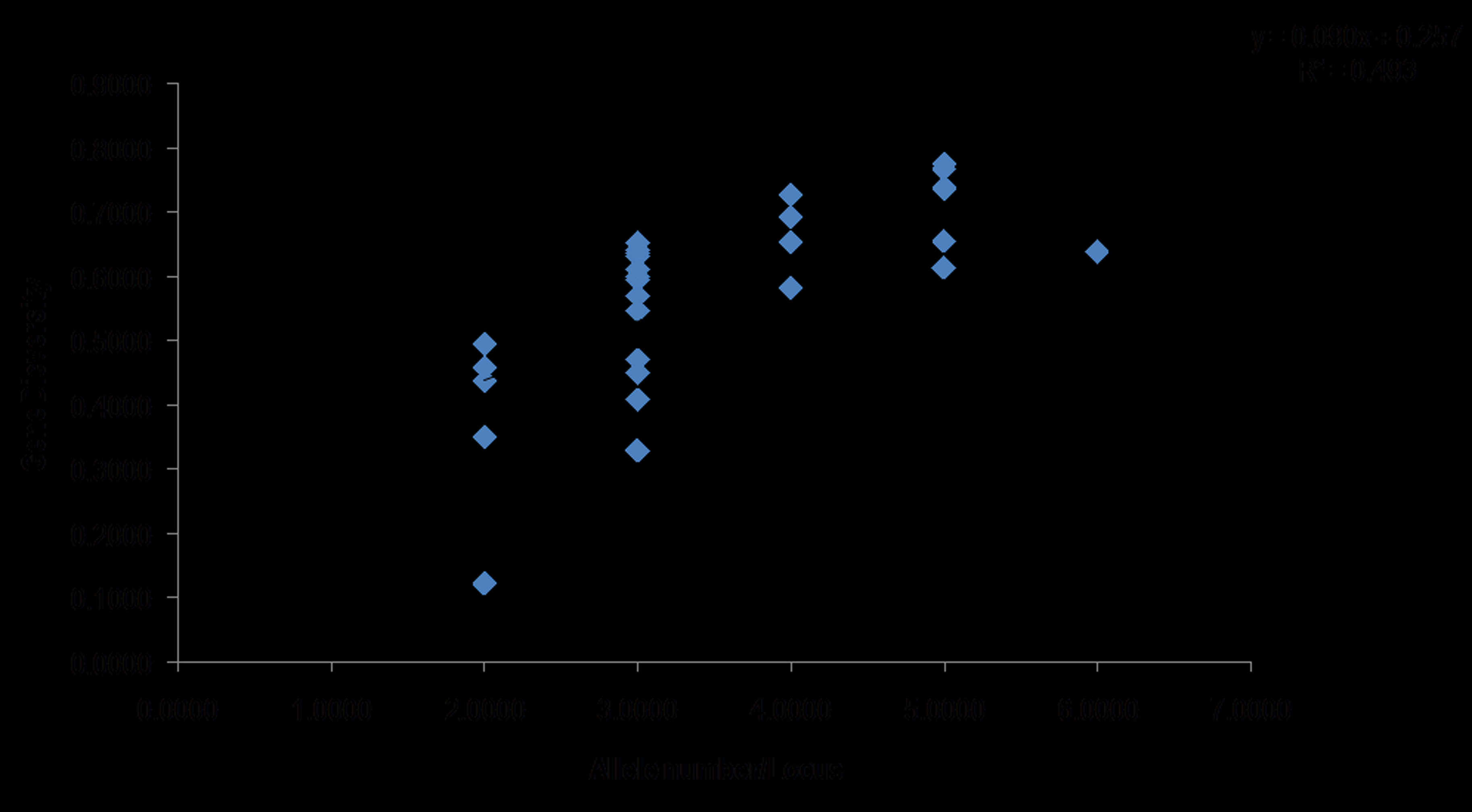

Supplement: S2 Fig — (TIF) [file pone.0156528.s002.tif]

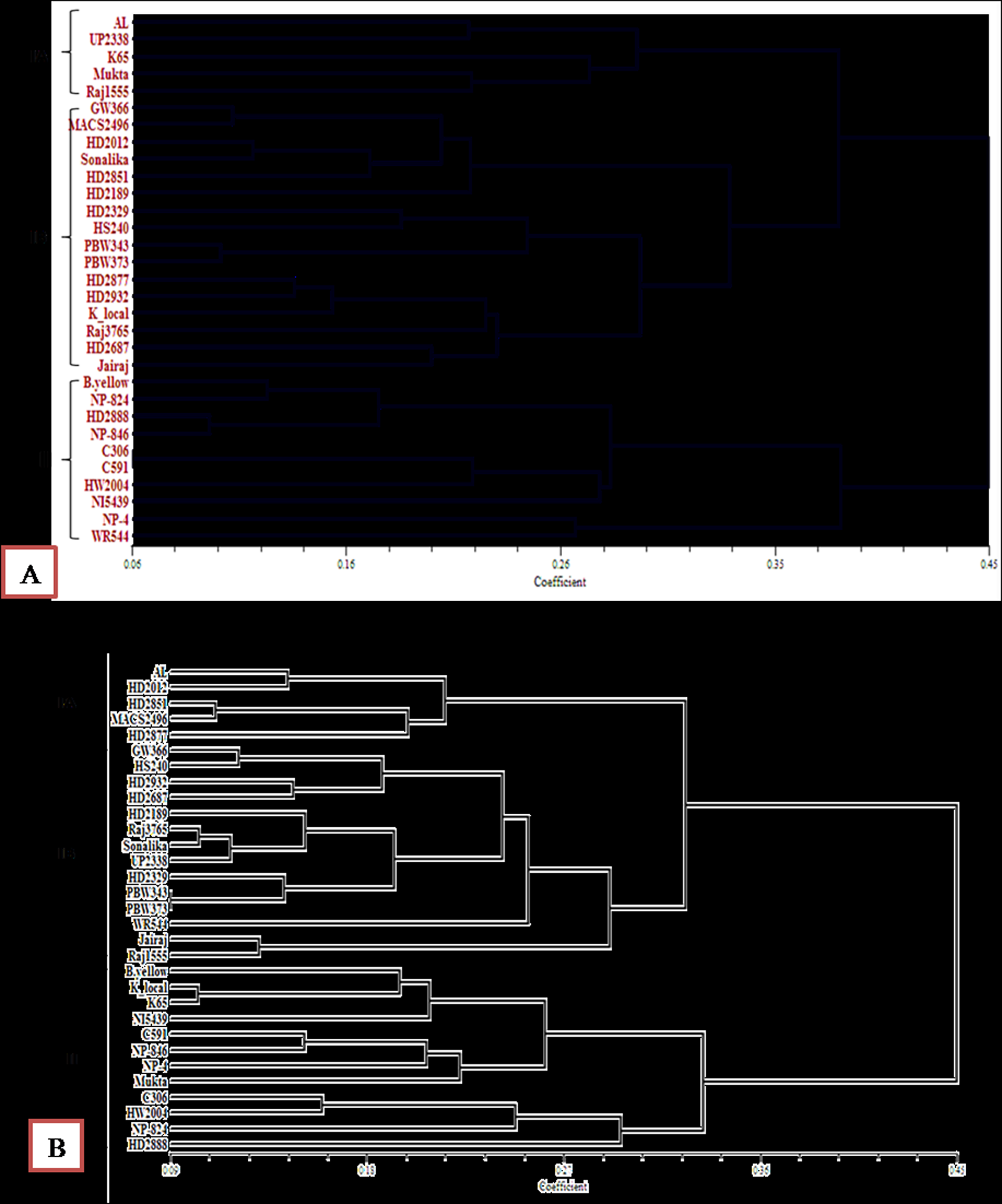

Supplement: S3 Fig — (TIF) [file pone.0156528.s003.tif]
