# Supplementary material for: Molecular and Morpho-Agronomical Characterization of Root Architecture at Seedling and Reproductive Stages for Drought Tolerance in Wheat
Source: PLoS One. 2016 Jun 9;11(6):e0156528. doi: 10.1371/journal.pone.0156528 (PMC4900657; doi:10.1371/journal.pone.0156528)
Supplement: S3 Table — (DOCX) [file pone.0156528.s006.docx]

| **S.No.** | **Name of Genotype** | **Year of release** | **Area of adoption** | **Conditions** | **Pedigree/parentage** |
| --- | --- | --- | --- | --- | --- |
| 1 | MUKTA | 1976 | CZ | TS,RF | HYB633I1GAZA(DR)/KPD25 |
| 2 | GW366 | 2006 | CZ | TS I | DL 802-3/GW 232 |
| 3 | C591 | 1934 | CZ | TS RF | TYPE9/8B |
| 4 | *B. Yellow | 1965 | KAR | TS RF | M. Local/Gaza |
| 5 | WR544 | 2003 | NW | LS I | KALYANSONA/HD1999// HD2204/DW38 |
| 6 | NP824 | 1960 | CZ | TS RF | WIS245”S”/NP165//NP770/3/C518/NP165 |
| 7 | NP4 | 1905 | NEPZ | TS RF | SEL.LOCAL MUNDIA |
| 8 | Agra Local | - | - | TS I | LV-Uttar-Pradesh |
| 9 | PBW373 | 1996 | NWPZ | LS I | ND/VG1944//KAL/BB/3/YACO”S”/4/VEE#5”S” |
| 10 | *JAIRAJ | 1980 | CZ | TS I | (T,POLO YAGULATE)PITATE-Z-B-W |
| 11 | UP2338 | 1994 | NWPZ | TS I | UP368/VL421//UP262 |
| 12 | HD2189 | 1979 | PZ | TS I | HD1963/H 01931 |
| 13 | K .LOCAL | - | - | - | - |
| 14 | *RAJ1555 | 1982 | CZ | TS I | COCORIT/RAJ911 |
| 15 | SONALIKA | 1935 | NW/NEPZ | LS I | MIDA-U/K117//B4946-A4-18-1/Y53//3*Y50 |
| 16 | NP846 | 1965 | NHZ | TS RF/LS I | RN/NP760 |
| 17 | HD2687 | 1999 | NWPZ | TS I | CPAN2009 / HD 2329 |
| 18 | PBW343 | 1995 | NWPZ | TS I | ND/VG1944//KAL/BB/3/YACO”S”/4/VEE#5”S” |
| 19 | K 65 | 1970 | ALL ZONES | - | KHARCHIA 65/ WL711 |
| 20 | HS240 | 1989 | NHZ | TS RF | AU//KAL/BB/3/BOW/PVN |
| 21 | HD2932 | 2007 | CZ,PZ | LS I | KAUZ/STAR//HD2643 |
| 22 | RAJ3765 | 1995 | NW/NEPZ | LS I | HD2402/VL639 |
| 23 | HD2329 | 1982 | NWPZ | TS I | SL”S”/NP852/4/PJ’P14//KT54B/3/K65/5/SKA/6/UP262 |
| 24 | HW2004 | 1995 | CZ | TS RF | C306*/7TR380-14*7/3AG14 |
| 25 | HD2877 | - | - | TS I | CDWR 9549/HD2347//HD2402 |
| 26 | C306 | 1965 | NWPZ/NEPZ | TS RF | RGN/CSK-3//2* C591/3/C21/N14//C281 |
| 27 | MACS2496 | 1991 | PZ | TS I | VEERY #5 |
| 28 | HD2012 | - | CZ | TS I | HD1467/HB208 |
| 29 | HD2888 | 2005 | NEPZ | TS RF | C306/*T.sphaerococcum*//HW2004 |
| 30 | HD2851 | 2003 | Delhi | TS I | CPAN3004/WR426/HW2007 |
| 31 | NI5439 | 1973 | PZ | TS I/RF | REP80/3*NP710 |

*****represents durum wheat genotypes, -= represents source not available; NWPZ=North Western Plane Zone, NEPZ=North Eastern Plane Zone; CZ=Central Zone; PZ= Peninsular Zone, NHZ= Northern Hill Zone; NW=North Western; TS=Timely sown; LS=Late sown; I=Irrigated; RF= Rain-fed.
