# Supplementary material for: Molecular and Morpho-Agronomical Characterization of Root Architecture at Seedling and Reproductive Stages for Drought Tolerance in Wheat
Source: PLoS One. 2016 Jun 9;11(6):e0156528. doi: 10.1371/journal.pone.0156528 (PMC4900657; doi:10.1371/journal.pone.0156528)
Supplement: S4 Table — (DOCX) [file pone.0156528.s007.docx]

**Table S4** Genotypes in various clusters based on 30 simple sequence repeat markers analysis

| **Cluster** | **Number** | **Reaction** | **Genotypes** |
| --- | --- | --- | --- |
| IA | 6 | DS | HD2877, HD2851, MACS2496, HD2012, HD2189, Bijaya Yellow |
| IB | 2 | DS | NP846, HD2932 |
| II | 4 | MS | PBW373, PBW343, K65, HD2329 |
| IIIA | 7 | DT | Mukta, C591, HW2004, C306, HD2888, NI5439, NP4 |
| IIIB | 2 | DT | NP824, HS240 |
| IVA | 6 | MT | WR544, Jairaj, Raj1555, Kharachiya Local, GW366, Agra Local |
| IVB | 4 | MT | Sonalika, HD2687, Raj3765, UP2338 |

DS=Drought sensitive; MS=Moderate Sensitive; DT=Drought tolerant; MT=Moderate tolerant
