# Supplementary material for: Molecular and Morpho-Agronomical Characterization of Root Architecture at Seedling and Reproductive Stages for Drought Tolerance in Wheat
Source: PLoS One. 2016 Jun 9;11(6):e0156528. doi: 10.1371/journal.pone.0156528 (PMC4900657; doi:10.1371/journal.pone.0156528)
Supplement: S7 Table — (DOCX) [file pone.0156528.s010.docx]

**Table S7.** Correlation among the different root and shoot traits analyzed in 31 genotypes of wheat grown in the PVC Pipes and field under drought stress condition at New Delhi

| Traits recorded in PVC pipes Traits recorded in field conditions | | | | | | | | | | | | | | | | |
| --- | --- | --- | --- | --- | --- | --- | --- | --- | --- | --- | --- | --- | --- | --- | --- | --- |
|  | | SH | RL | RL_SL | RM | SM | RM_SM | ETL | TTL | PH | TN | PL | SL | BYD | GYD | TKW |
| SH |  | 1 |  |  |  |  |  |  |  |  |  |  |  |  |  |  |
| RL |  | .770^**^ | 1 |  |  |  |  |  |  |  |  |  |  |  |  |  |
| RL_SL |  | .324 | .850^**^ | 1 |  |  |  |  |  |  |  |  |  |  |  |  |
| RM |  | .567^**^ | .648^**^ | .476^**^ | 1 |  |  |  |  |  |  |  |  |  |  |  |
| SM |  | .358^*^ | .519^**^ | .473^**^ | .813^**^ | 1 |  |  |  |  |  |  |  |  |  |  |
| RM_SM |  | .457^**^ | .275 | .008 | .382^*^ | -.176 | 1 |  |  |  |  |  |  |  |  |  |
| ETL |  | .114 | .075 | .025 | .074 | .120 | -.079 | 1 |  |  |  |  |  |  |  |  |
| TTL |  | .123 | .140 | .126 | .251 | .308 | -.035 | .523^**^ | 1 |  |  |  |  |  |  |  |
| PH |  | .639^**^ | .828^**^ | .702^**^ | .438^*^ | .456^**^ | .082 | -.066 | -.100 | 1 |  |  |  |  |  |  |
| TN |  | .677^**^ | .722^**^ | .501^**^ | .365^*^ | .125 | .425^*^ | .059 | .022 | .578^**^ | 1 |  |  |  |  |  |
| PL |  | .623^**^ | .750^**^ | .581^**^ | .368^*^ | .307 | .129 | .154 | -.023 | .796^**^ | .562^**^ | 1 |  |  |  |  |
| SL |  | -.164 | -.102 | -.031 | -.295 | -.104 | -.222 | -.148 | -.162 | .092 | -.063 | -.104 | 1 |  |  |  |
| BYD |  | .517^**^ | .476^**^ | .255 | .428^*^ | .212 | .348 | .111 | .049 | .468^**^ | .657^**^ | .494^**^ | .135 | 1 |  |  |
| GYD |  | .638^**^ | .631^**^ | .404^*^ | .323 | .118 | .322 | -.123 | .006 | .492^**^ | .826^**^ | .509^**^ | -.030 | .644^**^ | 1 |  |
| TKW |  | .713^**^ | .774^**^ | .557^**^ | .556^**^ | .385^*^ | .286 | .151 | .039 | .635^**^ | .741^**^ | .768^**^ | -.229 | .623^**^ | .704^**^ | 1 |
| SH=Shoot height; RL=root length; RL_SL=ratio of root length and shoot length; RM=root mass; SM=shoot mass; RM_SM= ratio of root ass and shoot mass; ETL=effective tillers per plant; TTL=total tillers per plant; PH= plant height; TN=number of tillers per plant; PL=peduncle length; SL=spike length; BYD=biological yield per plant; GYD=grain yield per plant; TKW=thousand kernel weight. ** and * represents correlation is significant at the 0.01 and 0.05 levels, respectively. | | | | | | | | | | | | | | | | |
